# The mechanism of formation, structure and physiological relevance of covalent hemoglobin attachment to the erythrocyte membrane

**DOI:** 10.1016/j.freeradbiomed.2016.12.024

**Published:** 2017-02

**Authors:** Elizabeth M. Welbourn, Michael T. Wilson, Ashril Yusof, Metodi V. Metodiev, Chris E. Cooper

**Affiliations:** a School of Biological Sciences, University of Essex, Colchester CO4 3SQ, UK; b Dept. of Exercise Science, Sports Centre, University of Malaya, 50603 Kuala Lumpur, Malaysia

**Keywords:** AE1, Anion Exchanger 1 (band 3 protein), BSA, (bovine serum albumin), CO, carbon monoxide, Co-IP, co-immunoprecipitation, DTT, dithiothreitol, Hb, hemoglobin, KCN, potassium cyanide, NEM, N-ethyl maleimide, PBS, phosphate buffered saline pH 7.4, Prx2, peroxiredoxin-2, SOSG, singlet oxygen sensor green, Erythrocyte, Hemoglobin, Membrane, Hemichrome, Oxidative stress, Blood storage, β Cys-93, Exercise, Senescence, Badwater ultramarathon

## Abstract

Covalent hemoglobin binding to membranes leads to band 3 (AE1) clustering and the removal of erythrocytes from the circulation; it is also implicated in blood storage lesions. Damaged hemoglobin, with the heme being in a redox and oxygen-binding inactive hemichrome form, has been implicated as the binding species. However, previous studies used strong non-physiological oxidants. *In vivo* hemoglobin is constantly being oxidised to methemoglobin (ferric), with around 1% of hemoglobin being in this form at any one time. In this study we tested the ability of the natural oxidised form of hemoglobin (methemoglobin) in the presence or absence of the physiological oxidant hydrogen peroxide to initiate membrane binding. The higher the oxidation state of hemoglobin (from Fe(III) to Fe(V)) the more binding was observed, with approximately 50% of this binding requiring reactive sulphydryl groups. The hemoglobin bound was in a high molecular weight complex containing spectrin, ankyrin and band 4.2, which are common to one of the cytoskeletal nodes. Unusually, we showed that hemoglobin bound in this way was redox active and capable of ligand binding. It can initiate lipid peroxidation showing the potential to cause cell damage. *In vivo* oxidative stress studies using extreme endurance exercise challenges showed an increase in hemoglobin membrane binding, especially in older cells with lower levels of antioxidant enzymes. These are then targeted for destruction. We propose a model where mild oxidative stress initiates the binding of redox active hemoglobin to the membrane. The maximum lifetime of the erythrocyte is thus governed by the redox activity of the cell; from the moment of its release into the circulation the timer is set.

## Highlights

• Hemoglobin binding to membranes initiated by mild oxidative stress.
• More binding of hemoglobin to membranes at higher oxidation state (Fe III to Fe V).
• Bound hemoglobin is redox active and retains ability to bind ligands.
• Bound complex contains spectrin, ankyrin and band 4.2.
• Bound complex is formed *in vivo* following extreme endurance exercise.

## 1 Introduction

Hemoglobin binding to the erythrocyte cell membrane has been implicated in senescence and the consequent targeting of the red cell for removal from circulation by macrophages *via* phagocytosis [1], [2], [3]. However, the molecular mechanism driving this binding and the consequent structure and reactivity of the bound complex are still a matter of debate [4], [5]. Models have been suggested whereby the binding of modified forms of hemoglobin (hemichromes) to the membrane interferes with the integrity of the cytoskeleton, resulting in clustering of Anion Exchanger 1 (AE1), the integral membrane protein more commonly known as band 3 [6]*. In vivo* evidence in favor of this model includes the finding of hemichromes and Heinz bodies on cell membranes from patients with unstable hemoglobin variants [7] and direct visualization of hemichrome/band 3 complexes [8]. *Ex vivo* it has been shown that pre-treatment of hemoglobin with strong oxidants such as phenylhydrazine or t-butyl hydroperoxide forms hemichromes which are seen to associate with membranes [6], [9] and separated membrane proteins such as band 3 [10].

As well as its importance in the life cycle of the erythrocyte *in vivo*, hemoglobin binding to the erythrocyte membrane likely plays a role in the storage lesions associated with blood marked for transfusion. During storage, damage to the cytoskeleton affects the flexibility of the cell with adverse rheological consequences. This is associated with hemichrome binding to membranes [11], decrease in the band 3 monomer and increases in band 3 degradation products [12] and a loss of vesicles containing aggregated hemoglobin band 3 [13].

Missing from these discussions is a coherent model as to how hemoglobin binding to the membranes is triggered and the molecular structure and reactivity of the bound complex. Part of the problem is the terminology of the hemoglobin species described. The term “hemichromes” in the scientific literature has been attributed to any unreactive low spin form of ferric (met) hemoglobin. Generally this is due to ligation of the distal histidine residue in the heme pocket, creating a bis-His iron complex, unable to react with external ligands. However, hemichrome formation can be irreversible or reversible [14]. We have previously shown that up to a quarter of the methemoglobin *in vivo* can be attributed to low spin heme iron [15], but that these species, either bis-His or Histidine/hydroxide, are in rapid equilibrium with the high spin reactive form of the protein. Detection of “hemichromes” associated with a membrane species therefore tells you very little about how the species was formed, or what chemical reactivity it might possess.

Probing the molecular mechanism of hemoglobin binding to the erythrocyte membrane is readily accessible to *in vitro* analysis. Unfortunately previous studies have used very strong oxidants such as phenylhydrazine. This does indeed induce membrane binding with associated hemichrome formation. However, phenylhydrazine is so reactive that it readily forms significant amounts of irreversible hemichrome species even in the absence of membranes. It is thus entirely possible that the hemichromes associated with the membrane are formed prior to the formation of the membrane-bound hemoglobin complex. This is less likely to occur when using milder physiological oxidants such as hydrogen peroxide.

Extreme exercise is a recognized method of causing oxidative stress *in vivo* [16] and triggering intravascular hemolysis [17]. It can therefore be used as a model of oxidative stress induced senescence. We therefore chose to study hemoglobin binding to the erythrocyte membrane using the physiological oxidant hydrogen peroxide and compared it to changes occurring following extreme endurance exercise. We show that the initiating species is high spin methemoglobin. The complex remains functionally and catalytically active even when it is bound to the membrane, and is able to initiate further oxidative damage. It increases following levels of endurance exercise that trigger erythrocyte clearance. We propose that this complex is a key intermediate in red cell senescence.

## 2 Experimental

### 2.1 Treatment of blood

Venous blood was drawn from healthy volunteers, using sodium citrate as anti-coagulant. Erythrocytes were separated by centrifugation at 2000 rpm for 15 min and used as a starting material to prepare purified hemoglobin or erythrocyte membranes (ghosts). Hemoglobin was prepared using the method of Antonini and Brunori [18]. Catalase was removed by anion exchange chromatography using DEAE Sephadex A50. Hemoglobin was stored as carbon monoxyhemoglobin at −20 °C. All hemoglobin concentrations in this paper are quoted in heme molar equivalents.

After washing in phosphate buffered saline (PBS) (150 mM NaCl; 2.5 mM KCl; 12 mM phosphate; pH 7.4) the membranes were isolated by hypotonic lysis using ≥10× volume 5 mM sodium phosphate pH 8.0 (lysing buffer) followed by washing in lysing buffer with centrifugation at 12,000 rpm for 20 min. A spectrum of the supernatant from 350 to 700 nm was taken using a Cary5 UV/vis spectrophotometer. The washing was repeated until there was no discernible peak in the supernatant spectrum. It took six or seven washes to remove all traces of hemoglobin from the optical spectra of the supernatant. This is in contrast to previous reports in the literature [6], [19], [20], [21], [22] that suggest using fewer washes to make hemoglobin-free ghosts. However, we found peaks from hemoglobin in the spectra even when the ghosts appeared white to the eye, thus requiring more washes to be sure of removing all unbound hemoglobin. All preparation work was carried out at 4 °C. The ghosts were re-suspended in PBS and either used immediately or stored at −80 °C.

### 2.2 Inducing hemoglobin binding to membranes

For studies in intact erythrocytes, washed packed cells were suspended in PBS (1:10) and subjected to oxidative stress by incubating with 5 mM NaNO_2_ at 4 °C. A spectrum taken after 25 min confirmed >95% conversion to methemoglobin. Samples were removed at intervals, the cells lysed and ghosts prepared as above. The oxidation of oxyhemoglobin by nitrite is complex with possible production of by-products such as nitrogen dioxide and hydrogen peroxide. We therefore used ferricyanide to convert oxyhemoglobin to methaemoglobin *in vitro* (see below), but as ferricyanide is not membrane permeable this was not possible for intact cell experiments.

For studies with the isolated hemoglobin, ghosts suspended in PBS with optical density (OD) 0.3–0.4 at 700 nm were incubated with 60 µM hemoglobin under different oxidation states or with different bound ligands at 20 °C for 30 min. They were then washed in lysing buffer as above. The washed ghosts were re-suspended in PBS to give OD 0.3–0.4 at 700 nm and a spectrum between 350 and 700 nm taken. To compensate for the effects of light scattering, 1st and 2nd derivatives of the spectrum were used to identify the position of the peak in the Soret region. To measure the peak height we used a three-point drop correction using 2 reference wavelengths [23]. Interfering absorbance at the peak wavelength was estimated using linear interpolation hence quantifying the hemoglobin present, using extinction coefficients from Antonini and Brunori [18]. Total protein present in the ghosts was measured using the Peterson modified Lowry method [24], [25].

The form of hemoglobin used for these studies was made from stock carbon monoxyhemoglobin with the identity of the final species being confirmed optically. Methemoglobin was formed by adding a few grains of potassium ferricyanide to 0.5–1.0 ml carbon monoxyhemoglobin (typically 2.0–2.5 mM) followed by flashing with bright light. Air was blown over the surface for a few minutes and the light treatment repeated. Excess ferricyanide was removed using a G25 Sephadex column with PBS as eluent. Methemoglobin, Fe(III), was converted to deoxyhemoglobin, Fe(II), by adding a few grains of sodium dithionite to the methemoglobin solution followed by passing down a second G25 Sephadex column to remove the excess dithionite, again using PBS as eluent. During the course of passing through the column, the deoxyhemoglobin combined with oxygen and formed oxyhemoglobin which was subsequently collected. Ferryl, Fe(IV) hemoglobin, was formed from methemoglobin by treating it with hydrogen peroxide (H_2_O_2_) in a 3:1 peroxide: heme ratio. Spectra were taken every 5 min and when the conversion to ferryl was >95% the reaction was stopped by the addition of a small amount of catalase to remove any unreacted peroxide. The reaction generally took between 15 and 20 min. When a study required access to the Fe(V) redox state (ferryl + protein radical), methemoglobin was treated *in situ* with a 2.5-fold excess of H_2_O_2_. Since the peroxide remains in the reaction mixture, a smaller excess is used compared to ferryl Fe(IV) preparation, in order to minimise exogenous damage. Cyanomethemoglobin was formed by mixing potassium cyanide and hemoglobin in a 2:1 ratio and the cyanomethemoglobin used immediately. The excess potassium cyanide was left in the solution to prevent the loss of the ligand during the incubation.

### 2.3 Changing redox states and ligand binding of bound hemoglobin

Hemoglobin was bound to ghosts as described and the bound hemoglobin was converted to different redox states. Ferrous (deoxy) hemoglobin was prepared by the addition of 19 µg/ml catalase and a few grains of sodium dithionite. Deoxy was converted to oxyhemoglobin *via* centrifugation washing under aerobic conditions. Deoxy was converted to carbon monoxyhemoglobin by incubation with CO, the buffers used for the subsequent washing steps also being saturated with CO. To attempt to induce the met (ferric) state potassium ferricyanide was titrated into the ghost suspension at a ratio of 2.5:1 (however, no further change was seen at concentration ratios up to 50:1). To form cyanohemoglobin, KCN (2:1 ratio) was added. For ferryl hemoglobin the bound hemoglobin was first converted to oxyhemoglobin followed by the addition of H_2_O_2_ (3:1).

### 2.4 Autoxidation

Hemoglobin was bound to ghosts with or without H_2_O_2_ as before. In half of the sample the hemoglobin was converted to oxyhemoglobin. The ghosts were incubated at 37 °C for 24 h with samples being removed for spectrophotometry at intervals. The ligand state of the hemoglobin present was identified from the position of the peak in the Soret region. Controls using isolated methemoglobin and oxyhemoglobin in the presence of clean ghosts were also incubated under the same conditions.

### 2.5 Lipid peroxidation

Prior to liposome addition, methemoglobin was bound to ghosts as described previously. Liposomes were formed by sonicating L-α-phosphatidyl choline from soybean type II-S (5 mg/ml) in PBS and then forcing through a Northern Lipids extruder using pressurized nitrogen (~25 bar). The liposomes were mixed with the hemoglobin-bound ghosts or with isolated methemoglobin at the same heme concentration. Singlet oxygen formation, a product of lipid peroxidation was followed using fluorescence from singlet oxygen sensor green (SOSG) (Molecular Probes) in a Fluostar Optima (BMG Labtech) fluorescence plate reader for 720 min. The rate of lipid peroxidation follows a sigmoidal-type curve. The rate of oxidative damage can be characterised by a lag phase and the maximal linear rate, with stronger oxidants decreasing the lag phase and increasing the maximal rate [26].

### 2.6 Kinetics of carbon monoxide recombination

Hemoglobin was bound to ghosts as before and the bound hemoglobin converted to deoxyhemoglobin. The ghosts were re-suspended in PBS at ~500 nM heme. CO was added and the mixture was placed in a sealed quartz cuvette. The CO was dissociated from the heme by a short flash (~10 ns) from a YAG laser (Applied Photophysics Ltd.) emitting at 532 nm and the recombination rate measured by taking absorption spectra 400–450 nm for 10 to 20ms. This was repeated at different CO concentrations. The change in absorption with time was fitted to a single or double exponential curve using SpectraKinetic Workstation v4.57.2 (Applied Photophysics Ltd). The curves were used to calculate the rate constants for the recombination reaction.

### 2.7 Electrophoresis and immunoblotting

Hemoglobin was bound to ghosts as described and the membrane proteins extracted using 0.25% Triton X-100 overnight at 4 °C. Insoluble components were removed by centrifugation and the protein solution concentrated by centrifugal filtration (Vivaspin microfiltration tubes 30 kDa cut-off). The samples were applied to 8% polyacrylamide gels with sodium dodecylsulphate (SDS) under non-reducing conditions using the method of Laemmli [27]. Generally two identical gels were run together. One was stained with Coomassie blue and the other used for Western blotting. The method used was similar to Towbin et al. [28] and Burnette [29]. The primary antibody was monoclonal antibody raised in mouse against whole human hemoglobin (Abcam).

### 2.8 Co-immunoprecipitation and mass spectrometry

Hemoglobin was bound to ghosts as described and the membrane proteins extracted using 0.25% Triton X-100 overnight at 4 °C. Insoluble components were removed by centrifugation. For co-immunoprecipitation (co-IP) the samples were pre-cleared with anti-human serum albumin antibody (Abcam) and protein A/G Plus-agarose beads (Santa Cruz Biotech) for 1–2 h. Beads were removed by centrifugation and anti-human hemoglobin antibody (Abcam) was added to the supernatant followed by agarose beads as before. After overnight shaking at 4 °C the beads were separated by centrifugation and washed three times with PBS.

Trypsin digestion and LC-MS/MS analysis was performed as previously described [30]. Briefly, washed agarose beads were incubated overnight at 30 °C with 20 µl trypsin solution containing 2.5 ng/µl sequencing-grade trypsin in 30 mM ammonium carbonate buffer, pH 8. The peptide samples generated by this digestion were separated by nano-scale LC and analysed online with a hybrid high-resolution LTQ/Orbtrap Velos instrument using electrospray ionization in positive polarity mode as previously described [30], [31], [32].

### 2.9 Involvement of thiol groups in binding

To determine the extent of disulphide bridges in the bound complex, ghosts were incubated with methemoglobin with or without the presence of 150 µM H_2_O_2_ and then washed exhaustively prior to the addition of the disulphide reducing agent, dithiothreitol (DTT). However, DTT was found to bleach the hemoglobin optical peak when the protein was bound to membranes. Therefore prior to the addition of DTT, hemoglobin was converted to the unreactive carbon monoxide (CO) bound form by the addition of 19 µg/ml catalase and a few grains of sodium dithionite, followed incubation with CO gas for a few minutes; the dithionite was subsequently removed by washing (centrifugation and removal of supernatant) with PBS saturated with CO. An optical spectrum of the ghosts confirmed conversion to carbon monoxyhemoglobin. The ghosts were re-suspended in PBS pH 7.8 saturated with CO, followed by incubation with DTT (5 mM) for 2 h at 20 °C. The ghosts were then washed 4 times in CO-saturated lysing buffer and finally re-suspended in PBS. The control sample received buffer with no DTT and the extent of hemoglobin binding to the membranes was compared.

Hemoglobin has a reactive thiol group on its β Cys-93 residue. To determine the effect of this residue on subsequent membrane binding its reactivity was blocked using pre-treatment with N-ethyl maleimide (NEM). The hemoglobin was assayed using p-(hydroxymercury) benzoate to confirm the removal of two reactive –SH residues per hemoglobin tetramer [33], [34]. The extent of membrane binding was studied by converting hemoglobin to the met (ferric) state using potassium ferricyanide and incubated with ghosts with or without 150 µM H_2_O_2_. These were then washed extensively and assayed as before.

### 2.10 *In vivo* exercise studies

Blood samples were analysed following a competitive international ultramarathon race and a separate two-day controlled exercise study with well-trained athletes. The Badwater Ultramarathon is a continuous 216 km one-stage race, with air temperatures up to 55 °C. Participants are as described by Yusof [35], [36]. In the 2 d exercise study 2 male volunteers (mean age 34.5; weight 72 kg height 175 cm) ran a two-staged 17mile run on consecutive days completing the course in 114 min on day 1 and 127 min on day 2 (mean times). Venous blood samples were taken pre-race, mid-race (9 miles into the run) and immediately after cessation of exercise. Subjects were kept well-hydrated during the entire study [36]. Data was averaged for the two individuals in all analyses.

Erythrocytes were separated and fractionated according to age using the Percoll density gradient method described by Salvo et al. [37] with minor modifications. Cell fractions were collected and washed with HEPES buffered isotonic saline. To verify the separation by age, cells from each fraction were lysed and passed through a mini-capillary column packed with Bio-Rex 70 cation-exchanger. Glycated hemoglobin was eluted with 5 mM sodium phosphate buffer pH 6.7 and the remaining hemoglobin eluted with buffer consisting of 50 mM sodium phosphate and 10 mM potassium cyanide (KCN) at pH 6.7. Hemoglobin concentration was measured spectrophotometrically at 415 nm and the ratio of glycated hemoglobin calculated. Levels of glycated hemoglobin were higher in the old cells (4.2±0.1%) than the young cells (1.7±0.1%), and remained constant throughout the study.

Membrane-bound hemoglobin was measured using the pyridine hemochromagen assay of Sanders et al. [38]. To quantify the spectrin present, ghosts were prepared from washed erythrocytes as described above and then treated with solubilisation buffer (125 mM Tris-HCl pH 6.8; 4% SDS; 20% glycerol; 10% 2-mercaptoethanol) and heat denatured at 95 °C for 5 min. Samples were then subjected to reducing SDS-PAGE (12% polyacrylamide) [27]. After staining the percentage of each protein present was measured using densitometric scans.

### 2.11 Enzyme activity in samples from *in vivo* exercise studies

Catalase activity in cells was measured using the method of Nicholls [39] in phosphate buffer (50 mM; pH 7.4; 37 °C). Purified catalase (Sigma) was used as a standard and the concentration was determined spectrophotometrically at 405 nm using an extinction coefficient of 4.44×10^5^ M^−1^ cm^−1^ for the tetrameric enzyme. Glutathione peroxidase activity was measured using a method of Flohe and Gunzler [40]. Glutathione reductase and reduced glutathione were incubated with lysed cells for 10 min at 37 °C in 100 mM phosphate buffer pH 7.0 with 1 mM EDTA. Following this incubation NADPH (1.5 mM in 0.1% sodium bicarbonate solution) was added. The reaction was initiated by the addition of t-butyl hydroperoxide solution and the consumption of NADPH monitored spectrophotometrically. The non-enzymic rate was assessed by substituting the sample with water. Mercaptosuccinate (100 µM) was used as an enzyme inhibitor to ensure the reliability of the measurement [41]. In order to measure the cell superoxide dismutase activity the cells were lysed and the hemoglobin was removed by mixing with an ethanol-chloroform (3:1) mixture [42]. The precipitate was removed by centrifugation and the SOD activity of the supernatant was measured using the method of Marklund and Marklund [43] with minor modifications. Supernatant was mixed with pyrogallol in a Tris-cacodylic acid buffer (50 mM; pH 8.2) with 1 mM EDTA and incubated at 25 °C for 10 min and the reaction terminated by the addition of hydrochloric acid. Activity was followed spectrophotometrically and compared with a commercially available human erythrocyte SOD (Sigma).

### 2.12 Statistics

Unless otherwise noted, data are presented as mean±SD. Unpaired two-tailed *t*-tests were used for comparison of bound hemoglobin reactivity compared to controls.

## 3 Results

### 3.1 Oxidative stress binding of hemoglobin to membranes *in vitro*

Erythrocytes have some bound hemoglobin with no external treatment (1.3±0.3% of total membrane protein, n=3). When treated with nitrite for 25 min at 4 °C to convert the hemoglobin to the ferric state the cells showed a nearly 10-fold increase in hemoglobin binding (15.0±0.3% of total membrane protein, n=3) compared to cells containing ferrous hemoglobin. Removal of nitrite and a subsequent incubation at 20 °C for up to 90 min did not result in any increase in this number.

Erythrocyte ghost membranes were made following cell lysis and high-speed centrifugation, free hemoglobin solution being removed from the supernatant following each centrifugation cycle. Ghosts were incubated with hemoglobin bound to different ligands or in different oxidation states. There was no significant difference between the amount of hemoglobin present in the membranes after incubation with oxyhemoglobin or the redox inactive states of carbon monoxyhemoglobin or cyanohemoglobin (Table 1). However, with redox active forms of the protein, the higher the oxidation state accessible, the greater the increase seen in the amount of hemoglobin bound, with 6.9 times more for ferric (III), 29 times more for ferryl (IV) and 120 times more for ferryl hemoglobin with added hydrogen peroxide (Fe(V)) compared to oxyhemoglobin.

Most experiments were done at pH 7.4 to mimic conditions found *in vivo*. As oxidative stress can potentially lower, and varying the solution pH can inform on mechanism, we compared membrane binding at pH 6.0 and pH 8.0. When comparing pH 6.0 to pH 8.0, acidification induced no change in the reactivity of carbon monoxyhemoglobin; a 1.4 fold-increase with methemoglobin (p<0.01), and a 2.0 fold-increase with ferryl hemoglobin (p<0.001).

### 3.2 Functional state of the bound complex *in vitro*

**Fig. 1:**
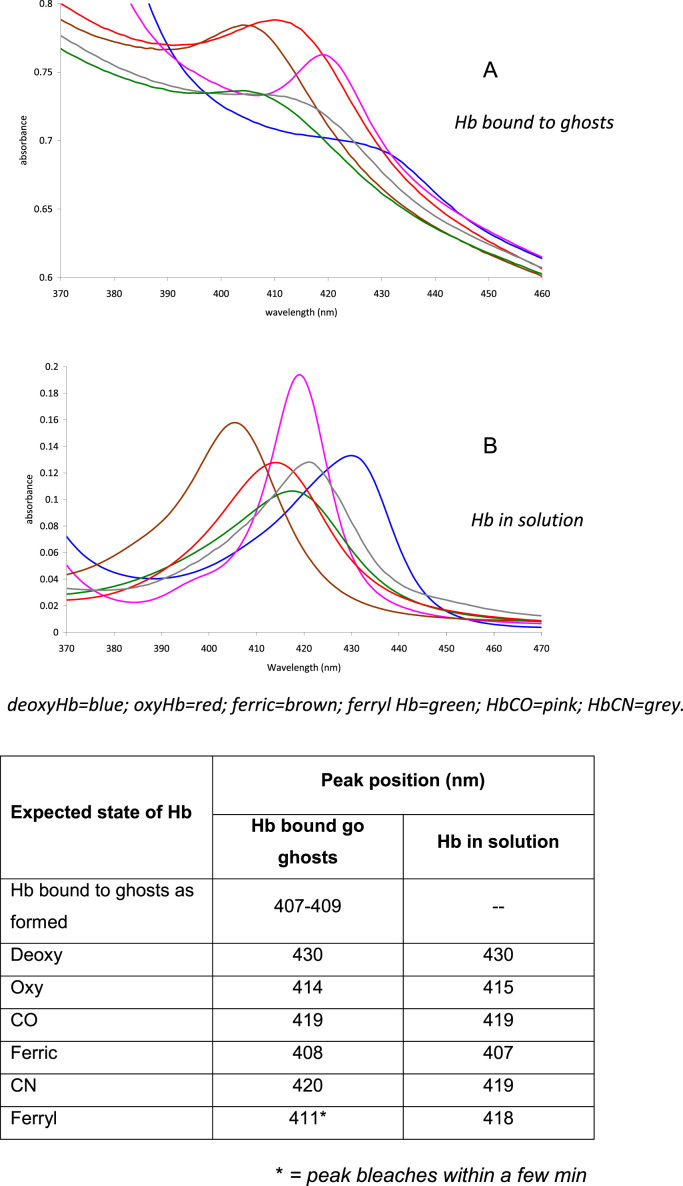
Spectra in Soret region of Hb (A) bound to ghosts and (B) in solution with different oxidation states or ligands. Hb bound to ghosts or in solution was treated as shown in Methods section to produce different oxidation states or bound ligands.

Hemoglobin bound to the ghost membrane can alter its redox state and bind ligands (Fig. 1). The position of the peak in the spectrum of bound hemoglobin suggests that when formed it is present in the ferric state. This was confirmed by the lack of any effect on the peak position following the addition of the oxidant potassium ferricyanide. Addition of sodium dithionite produced ferrous (deoxy) hemoglobin, which readily converted to oxyhemoglobin when the dithionite was washed out. Addition of CO to bound ferrous hemoglobin and potassium cyanide to bound ferric hemoglobin gave peaks with the same wavelengths as carbon monoxyhemoglobin and cyanohemoglobin in solution respectively. It was not possible, however, to demonstrate the formation of ferryl hemoglobin. The addition of hydrogen peroxide instead resulted in a spectral peak at 410–411 nm, possibly indicating the formation of hemichromes.

**Fig. 2:**
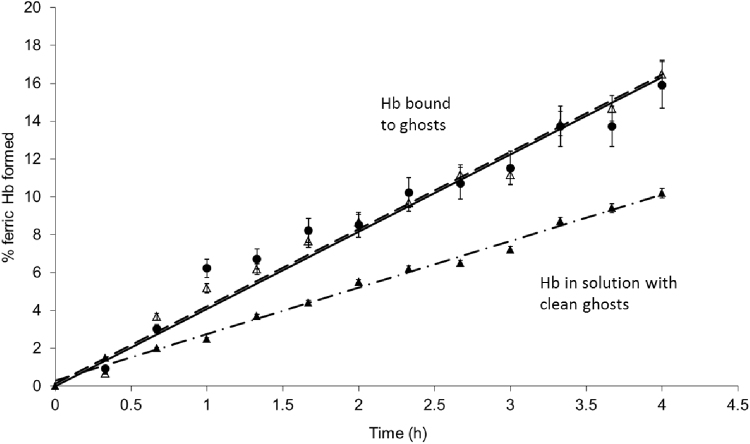
Rate of autoxidation of Hb bound to ghosts. Hb bound to ghosts using ferric Hb with (closed circles – dashed line) or without (open triangles – solid line) hydrogen peroxide was converted to the oxy state. These ghosts were incubated at 37 °C and the ferric Hb formed was assayed spectrophotometrically. These 2 lines are overlaid. OxyHb in the presence of clean ghosts was also used (closed triangles – dashed and dotted line). *Mean*±*SD (n=3).*

Oxyhemoglobin incubated at 37 °C spontaneously converts to the ferric form, a process termed autoxidation. Hemoglobin bound to ghosts and then converted to the oxy form autoxidises at a faster rate compared to free oxyhemoglobin in the presence of ghosts (Fig. 2). Whilst methemoglobin left in solution did not alter during 24 h treatment at 37 °C, methemoglobin bound to ghosts altered its Soret absorbance peak from 407 nm to 410 nm. This is similar to the change seen when H_2_O_2_ was added to ghosts with bound hemoglobin, and is consistent with a slow conversion to a hemichrome state over time.

**Fig. 3:**
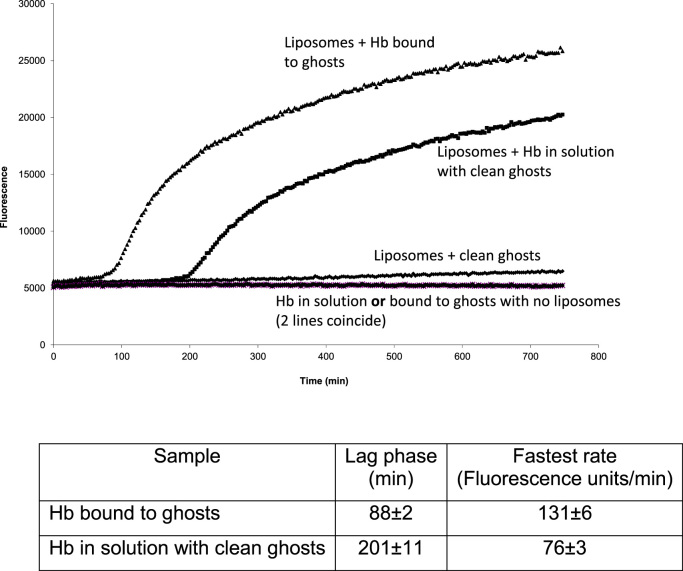
Lipid peroxidation initiated by Hb bound to ghosts or in solution. Hb bound to ghosts or in solution was added to liposomes with or without clean ghosts ([Hb]=560 nM). Lipid peroxidation was followed using fluorescence produced by the action of singlet oxygen on SOSG. The time shown is after the addition of SOSG. Mean values±SD; (n=4).

Hemoglobin in solution can initiate lipid oxidation, the autocatalytic cycle leading to a cascade of peroxidative reactivity [44]. We found no evidence of lipid peroxidation in the ghost membranes containing the bound hemoglobin (Fig. 3), presumably because of the presence of endogenous antioxidants. However, when exogenous, antioxidant free liposomes were added lipid peroxidation did occur. The lag phase was shorter and the rate faster than that for the same concentration of free hemoglobin added to a mixture of liposomes and clean ghosts. Binding to the ghost membranes therefore increases the oxidative reactivity of hemoglobin.

Carbon monoxyhemoglobin is stable in the dark, but light can dissociate the CO ligand. Measuring the rate of CO recombination to the heme is a sensitive measure of the dimer/tetramer equilibrium [81], [82]. At the low concentrations used in these experiments (500 nM), essentially all the free hemoglobin would be expected to be present as a dimer. This has a fast rate of CO recombination. Using flash photolysis to follow the reaction, hemoglobin in solution did indeed show a single, fast rate of CO recombination. The hemoglobin tetramer has a slower rate of CO recombination than the dimer. Therefore when the hemoglobin concentration is increased a second slower rate can be measured, the proportion being related to the dimer/tetramer ratio.

**Fig. 4:**
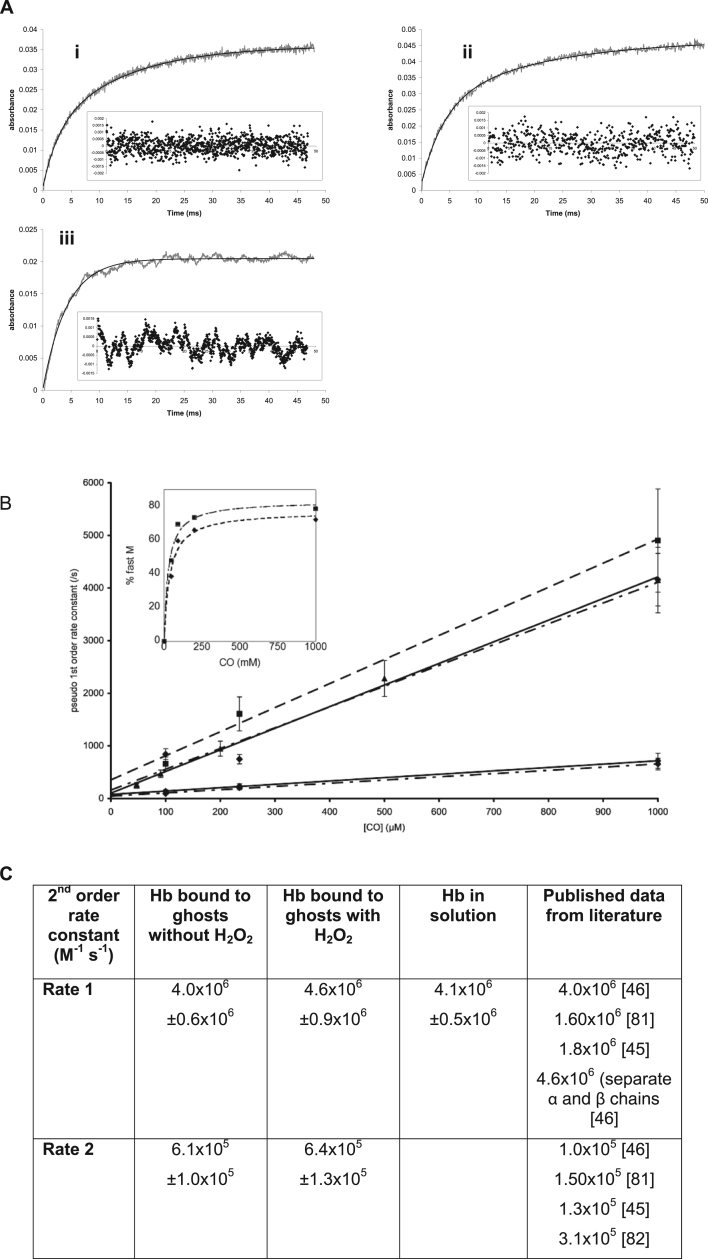
Kinetics of CO concentration dependence for recombination of CO to Hb bound to ghosts and in solution. A) Examples of time courses of CO recombination fitted to exponential curves – Hb bound to ghosts (i) without and (ii) with hydrogen peroxide present in the binding incubation; (iii) Hb in solution. (Hb conc=500 nM) (i) and (ii) are fitted to double exponential curves, (iii) is fitted to a single exponential. Insets show residuals from the fitted curve. B) Using exponential curve fits from A, pseudo 1st order rate constants were calculated and plotted against CO concentration. Hb bound to ghosts with H_2_O_2_=dashed line; Hb bound to ghosts without H_2_O_2_=dashed and dotted line; Hb in solution=solid line. Mean±SD; (n=4). Inset shows the variation of % of fast rate component with CO concentration with a hyperbolic fit approaching the asymptote of 100% dimer at very high CO. C) The gradients from the graph (B) of pseudo 1st order rate constants show the rate constants for the recombination of CO to Hb. These were compared with published data. Mean rate±SD; (n=6).

However, using a solution of hemoglobin-bound ghosts at the same (500 nM) heme concentration the recombination data fit best to a double exponential curve (Fig. 4A). The rates of both reactions were CO concentration dependent. The fitted curves were used to calculate the rate constants for the fast and slow phases (Fig. 4B). The fast phase for bound hemoglobin gave the same rate as for dimeric hemoglobin in solution (Fig. 4C), both rates being similar to published literature values. The slow rate is of the same order of magnitude as that for the binding of CO to hemoglobin tetramers in solution. Hemoglobin bound to membranes appears therefore to be able to undergo genuine dimer/tetramer interconversion. As with isolated hemoglobin in solution [45], [46] the proportion of the fast (dimer) phase increased relative to the slow (tetramer) phase as the CO concentration was increased (Fig. 4B) confirming a genuine interconversion between dimers and tetramers, not just two different forms of bound hemoglobin species that do not interact.

### 3.3 Chemical nature of the bound complex *in vitro*

**Fig. 5:**
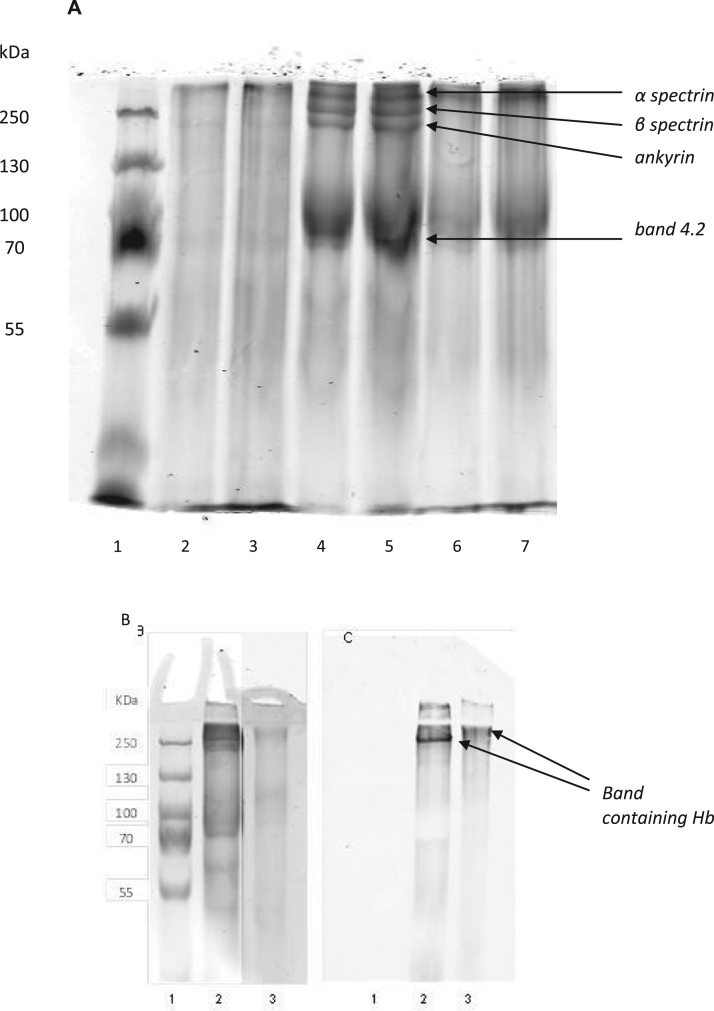
SDS-PAGE and WB of proteins extracted from membranes with after incubation with Hb followed by extraction with 0.25% Triton X-100. (A) SDS-PAGE with duplicate lanes. Lane 1=m.w. marker; lanes 2 and 3=extract from ghosts not incubated with hemoglobin; lanes 4 and 5=extract from ghosts incubated with ferric hemoglobin; lanes 6 and 7=extract from ghosts incubated with ferric hemoglobin and hydrogen peroxide. Bands corresponding to band 4.2 (72 kDa), ankyrin (205 kDa), β spectrin (250 kDa) and α spectrin (280 kDa) can be seen in lanes 4 and 5. Definition of the ankyrin and spectrin bands is lost in lanes 6 and 7. Very little membrane protein is extracted from membranes without bound Hb using 0.25% Triton X-100 (Lanes 2 and 3). (B) SDS-PAGE as in (A) and (C) corresponding Western blot using anti-Hb. Lane 1=mw marker; lane 2=extract from ghosts incubated with ferric hemoglobin; lane 3=extract from ghosts incubated with ferric hemoglobin and hydrogen peroxide. Bands containing Hb can be seen at about 250 kDa in lane 2 and higher in lane 3. Gel lane 3 is viewed at higher contrast to visualise the bands more clearly. (Mw in kDa shown on left hand side).

Proteins in membranes that had been incubated with hemoglobin were extracted using Triton X-100 and then subjected to SDS-PAGE under non-reducing conditions (Fig. 5A). No bands can be seen from ghosts that had not been incubated with any external hemoglobin, since the concentration of Triton X-100 is not high enough to extract the membrane proteins from undamaged cells. The presence of bands in the other lanes shows that there is an effect on the membrane proteins from hemoglobin binding. Bands corresponding to spectrin, ankyrin and band 3 can be seen for ghosts incubated with methemoglobin, Fe(III). Immunoblotting using anti-hemoglobin antibodies showed the presence of hemoglobin in a band in the region of 250 kDa (Fig. 5B). Similar treatment of ghosts incubated with methemoglobin and H_2_O_2_ forming Fe(V) gave a band consistent with band 3, but some higher molecular weight bands were missing. Immunoblotting showed hemoglobin present at a slightly higher molecular weight than seen when only methemoglobin was used in the original incubation. Since it is not fully reliable to identify proteins purely by their molecular weight co-IP and mass spectrometry was used to confirm the identity of proteins present in the hemoglobin-containing complexes.

Membranes were incubated with hemoglobin and the proteins were extracted using Triton X-100. The extract was treated using co-IP with anti-hemoglobin antibody. The extracted proteins were subjected to mass spectrometry. The protein complex extracted from membranes treated with hemoglobin in the ferric state contained the membrane-associated proteins α and β-spectrin, ankyrin and band 4 protein with α and β-hemoglobin (Table 2). The protein complex present after membranes had been treated with ferric hemoglobin and H_2_O_2_ showed only significant amounts of β-hemoglobin, and no other protein associated with hemoglobin. The protein complexes stay intact despite extensive washing, extraction using detergent and electrophoresis under denaturing conditions. This suggests that the interaction is very strong, presumably covalent bonding.

The importance of disulphide bridges in hemoglobin binding to membranes was investigated in two ways. Reactive thiol groups on hemoglobin (predominantly βCys-93) were blocked using NEM before incubation with ghosts. This resulted in a significant decrease in binding to the membrane when using methemoglobin and H_2_O_2_ to initiate the reaction (down 34±4% (p<0.001)). Forming the bound hemoglobin in the absence of NEM, and then treating the membranes with DTT to break any disulphide bonds, resulted in the removal of 30±2% (p<0.002) of bound hemoglobin. Both these results suggest that about a third of the bound hemoglobin is associated with disulphide bridge formation. In the case of methemoglobin alone the result is somewhat larger; NEM dropped the reaction by 46±1% (p<0.001) and DTT removed 56±2% (p<0.01) of the hemoglobin bound in the absence of NEM.

### 3.4 Using extreme exercise to bring about oxidative stress *in vivo*

**Fig. 6:**
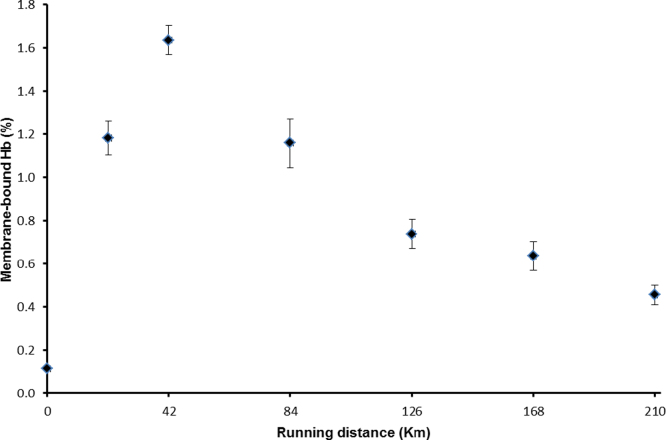
Membrane-bound Hb during the Badwater Ultramarathon. Mean±SD; n=5.

In order to investigate the mechanism of oxidative stress *in vivo* we used an extreme exercise regime. The Badwater Ultramarathon is a continuous 216 km race starting in Death Valley, California with a total of 4000 m ascent and 1400 m descent. Air temperatures reached 55 °C during the race which takes place annually in July. The time limit for finishing the race set by the organisers is 60 h, our volunteers finishing in 48.25±9 h. All participants in the study were trained males with experience of running ultramarathons. Erythrocyte membranes from pre-race blood samples show very low levels of bound hemoglobin. This increased during the first 42 km followed by a steady decline to the end of the race. Post-race levels were higher than at the start (Fig. 6).

**Fig. 7:**
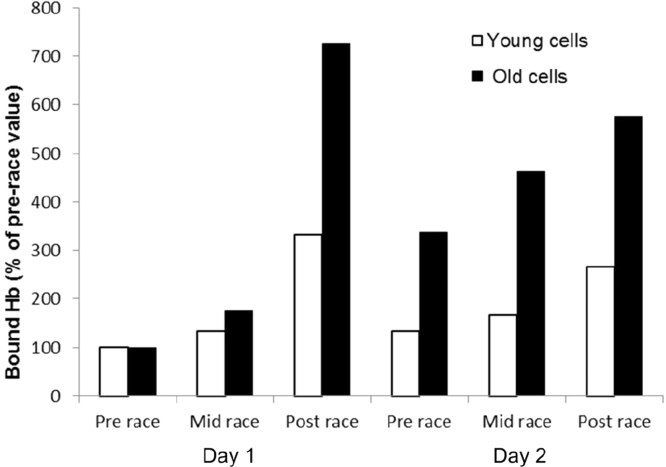
increase in membrane-bound Hb in age-fractionated cells. Membrane-bound Hb in red cells taken before, midway and after running 17 miles on consecutive days as % of pre-race values.

In order to explore further the time course of Fig. 6a more detailed analysis was performed on a case study in two well-trained individuals who undertook two 17mile runs on consecutive days, completing the course in average times of 114 min on day 1 and 127 min on day 2. Blood samples were taken at the start, at mile 9 and after each run. Erythrocytes were separated into age fractions by density. On day 1 the ratio of young:old cells was 0.76±0.06 and on day 2 it was 1.0±0.03. There was an increase in membrane-bound hemoglobin during the runs (day 1=10 fold increase; day 2=8 fold increase compared to original value) with the starting level higher on day 2. Analysis of age-fractionated cells showed the level of bound hemoglobin in young cells is lower compared to old cells. There was an increase in bound hemoglobin in both age fractions during the runs, with a higher starting level on day 2, but the old cells showed a larger percentage increase (young cells 3.3 fold and 2.7 fold increase; old cells 7.3 fold and 5.8 fold increase on day 1 and day 2 respectively) (Fig. 7).

**Fig. 8:**
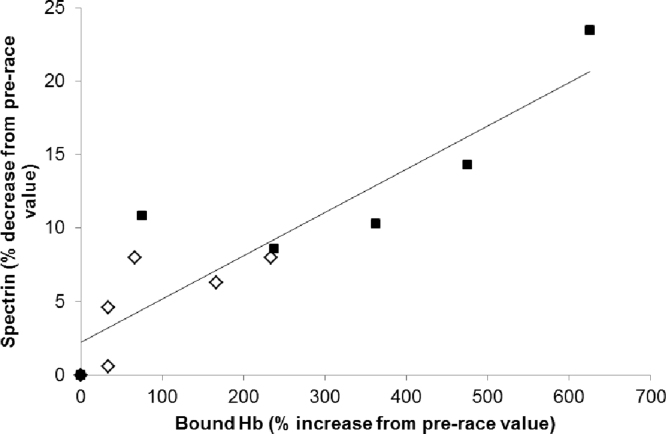
Correlation between increase in membrane-bound Hb and decrease in membrane spectrin. Blood samples were taken before, midway and after running 17 miles on consecutive days. Values given are the change compared to pre-race values. Young cells shown as open diamonds; old cells shown as closed squares. R=0.90; p<0.0001.

The membrane protein spectrin decreased by 10% after day 1% and 9.5% after day 2 compared to pre-race values, with some recovery overnight. There is a difference between old and young cells. At the start of day 1 old cells contained 98% of the spectrin seen in young cells. Spectrin in young cells had decreased by 8% at the end of day 1 and by 6% at the end of day 2, whilst in old cells it decreased by 23% and 14% on the two days. There is a very strong correlation between the increase in bound hemoglobin and the decrease in spectrin (Fig. 8) with a Pearson correlation coefficient of 0.90 (p<0.0001).

## 4 Discussion

This study demonstrates that covalent binding of hemoglobin to the erythrocyte is a consequence of oxidative stress. The hemoglobin initially remains in a redox active functional form, reverting with time to the irreversible dysfunctional hemichrome complexes.

The strong oxidant phenylhydrazine has been used previously to probe hemoglobin oxidative damage [6], [47], [48], [49], [50], [51], [52]. Phenylhydrazine and its derivatives react with oxyhemoglobin forming reactive intermediates [53]; a hydroxyl radical is released in the correct position to hydroxylate a methene bridge in the heme group and phenyldiazene becomes liganded to the heme in the 6th position [54]. Thus, phenylhydrazine brings about damage to hemoglobin leading to hemichrome formation but the mechanism does not mimic any reactions seen normally *in vivo*. Therefore we chose the mild membrane permeable oxidant nitrite to initiate methemoglobin formation in intact cells. Nitrite has the advantage that it is unlikely, in and of itself, to induce oxidative stress or covalent binding of proteins to membranes (whilst maintaining the capacity to do this indirectly *via* triggering oxidative reactions in heme proteins). It is also present in the erythrocyte under physiological conditions [55]. In the isolated erythrocyte, converting the hemoglobin to the met (ferric) form using nitrite caused a significant binding of the protein to the membrane, resulting in hemoglobin contributing to almost 1/6th of the total membrane protein content. Longer incubations did not increase this proportion, suggesting specific saturable sites of attachment to the membrane.

On its own, methemoglobin can redox cycle in the presence of lipids, initiating lipid peroxidation and hemoglobin breakdown [44], [56]. Reactive nitrogen species can also interact with hemoglobin forming nitrosyl complexes with the heme [57] or protein [58]. It is therefore possible that nitrite itself, a nitrite derivative, or a nitrite modified hemoglobin could be required to initiate hemoglobin binding to the erythrocyte membrane. However, our *in vitro* studies argue against this as using the oxidant ferricyanide to form ferric hemoglobin still showed significant membrane binding. Therefore nitric oxide, nitrite or a related reactive nitrogen species are clearly not a pre-requisite for heme binding to the erythrocyte membrane.

The extent of hemoglobin binding correlates with the oxidative reactivity of the hemoglobin. Converting the ferric Fe(III) state to the ferryl Fe(IV) form increased binding. Forming Fe(V) by adding peroxide *in situ* resulted in a further increase. Some of this may be due to direct reaction between peroxide and membrane proteins, but since the peroxide concentration is not high, we believe that the major effect is due to the formation of ferrylhemoglobin with a radical on the protein moiety. Others have shown increased bound hemoglobin with a decreased antioxidant capacity after peroxide addition [59]. Lowering the pH, a condition known to enhance the reactivity of ferryl hemoglobin [60], also increased binding. The bound hemoglobin is initially fully functional, able to react with oxygen and form a functional quaternary complex that can undergo reversible dimer/tetramer interconversion. Bound hemoglobin is also redox active, the ferrous Fe(II) oxy form showing enhanced autoxidation rate, converting to the Fe(III) state over time. Once formed on the membrane this Fe(III) state shows enhanced pro-oxidative activity compared to free hemoglobin. In the process the Fe(III) state converts to an unreactive form consistent with the bis-histidine iron ligation found in hemichromes. It is therefore clear from these results that, in direct contrast to previous suggestions [3], [5], [21], hemichromes are a *consequence* of hemoglobin binding to the erythrocyte membrane, not the cause of that binding.

Hemoglobin in the deoxy state has been shown to bind reversibly to the cytoplasmic domain of band 3 [61] potentially switching energy metabolism between the glycolytic and pentose phosphate pathways [62], [63]. *In vivo* band 3 associates with other membrane proteins [64], [65]. Using a mild oxidative stressor (metHb) we showed that hemoglobin binds in a high molecular weight complex containing spectrin, ankyrin and band 4.2 with both α and β hemoglobin present. These three membrane proteins are present in one of the band 3 nodes of the erythrocyte membrane [66], [67]. With more severe oxidative stress (ferryl hemoglobin) hemoglobin was found in an even higher molecular weight complex with a loss of definition of spectrin and ankyrin bands. When the complex was extracted using co-immunoprecipitation it contained only β-hemoglobin, and no other membrane proteins in significant amounts. There is a significant role for sulphydryl bonds, presumably *via* the β–93Cys, although under higher oxidative stress, the role of -SH reactivity was decreased.

Band 3 binds reversibly to deoxyhemoglobin *via* the bisphosphoglycerate cleft [61]. Deoxyhemoglobin is predominantly in the T state conformation. A fraction of methemoglobin can also adopt this T state [68], [69], [70]. A range of amino acids in hemoglobin can be oxidatively modified (*e.g.* βCys-93, βCys-112, βTrp-15, β-Trp55, β-Tyr145 [71]). A plausible interpretation of the mechanism of binding is therefore that an oxidative event occurs when T state methemoglobin is transiently bound to band 3, forming irreversible bonds including, but not exclusively, the β−93Cys. Under conditions of higher oxidative stress, the damage is more chaotic, with covalent bonding being initiated between membrane proteins, but not necessarily involving hemoglobin.

An unusual feature of the covalently bound complex is that it can apparently undergo dimer/tetramer interconversion. This places some limits as to its structure. Band 3 comprises 25% of the integral membrane proteins of the erythrocyte ghost [72]. When bound in the ferric state only 0.5% of the total protein is hemoglobin, measured per heme. Therefore on average 1% of the 6×10^5^ band 3 dimers/cell [72] have a dimer of hemoglobin bound. Making the assumption that these are evenly distributed in the membrane, adjacent band 3-hemoglobin complexes are separated by 0.1 µm. Applying Einstein's equation of diffusion with the diffusion coefficient of 1.4×10^–11^ cm^2^ s^−1^[73] it would take more than one second for the band 3 to diffuse this distance. Even allowing for some clustering of the hemoglobin-bonded band 3 chains this is still well beyond the time-scale of the carbon monoxide recombination reaction. Therefore if the hemoglobin were bound as a dimer there would not be enough time for two molecules to collide and form a tetramer during the flash photolysis experiments. It appears most likely that all four subunits of hemoglobin are attached to the membrane together and can undergo partial subunit dissociation, yet remain tethered to the membrane during this process. This situation is not as unusual as it might appear. Chemically cross-linking hemoglobin with 2- nor-2-formylpyridoxal 5′-phosphate, a bifunctional derivative of pyridoxal 5′-phosphate, yields a species that behaves in part as tetramer and in part as dimer, in the latter case the two αβ dimers remain chemically linked but functionally independent [74].

If the oxidative protection of the cell becomes overwhelmed then the hemoglobin cannot be converted back to the ferrous form quickly enough and there is a possibility of it binding to membrane proteins. The anti-oxidant enzymes that protect the erythrocyte from oxidative damage decrease as cells age [75]. Consequently it would be expected that older cells are more likely to succumb to the damage from oxidative stress since they have fewer protective enzymes present. In this model oxidative stress triggers hemoglobin binding to cell membranes throughout the life cycle of the cell, but this becomes increasingly significant as the cell ages. The membrane complex, being redox active, likely in and of itself increases oxidative stress and membrane damage, leading ultimately to hemichrome formation and removal from the circulation. It is not the actual age of the cell that brings about removal but the oxidative damage; increasing oxidative stress effectively accelerates the ageing process.

Extreme exercise is a good model system to test this hypothesis as it triggers increases in both the oxidation of hemoglobin [16] and erythrocyte hemolysis [17]. The ultramarathon study (Fig. 6) shows a rapid increase in membrane bound hemoglobin at the start of the race consistent with an oxidative stress trigger as seen *in vitro*. There is a subsequent decrease though it never falls to pre-exercise levels. The secondary decrease could be explained if the hemoglobin in younger cells is less likely to bind to membranes following *in vivo* oxidative stress and that during the course of the ultramarathon the average age of the population decreases. This is consistent with previous findings that erythrocyte destruction during extreme exercise is biased towards old cells [17].

This was confirmed in our two-day endurance exercise study. Here the average age of the erythrocytes in circulation decreased as older cells were cleared overnight and the increase in hemoglobin binding to the erythrocyte cell membranes did indeed occur preferentially in the older cells (Fig. 7). This vulnerability of older cells to oxidative stress and subsequent hemoglobin binding to membranes and hemolysis is consistent with the decrease in antioxidant enzyme activity observed.

We have previously shown that oxidative hemolysis induced by extreme endurance exercise is linked to alterations in the membrane protein spectrin [35]. In the present study we see decreasing levels of the 250–280 kDa spectrin bands following extreme exercise. This loss of detectable spectrin *in vivo* correlates with the formation of membrane bound hemoglobin (Fig. 8). Our *in* vivo results are therefore consistent with the formation of the covalent hemoglobin:spectrin complex we see *in vitro.*

It would be interesting in future studies to compare the interaction of other oxidative stress markers with the binding of hemoglobin to the erythrocyte membrane. Peroxiredoxin-2 (Prx2) is a protein that is abundant in the erythrocyte [76]. It catalyses the breakdown of hydrogen peroxide by the formation of a dimer through disulphide bonds, the sulphydryl groups being regenerated by reduction by thioredoxin. The protective role of Prx2 is expected to reduce with cell age as both the Prx2 and thioredoxin (and other enzymes required for its reduction) will also be depleted. Prx2 binds to cell membranes competitively with hemoglobin under oxidative stress [59], [77] and in stored cells with ageing [78]. *In vivo* Prx2 should therefore protect against hemoglobin-induced oxidative damage to the membrane by removing hydrogen peroxide (and hence Fe(IV) and Fe(V) heme) and by occupying sites where hemoglobin can bind. However, once bound Prx2 ceases to be functional as an antioxidant, since the sulphydryl groups required for its antioxidant activity are also likely required for its membrane-binding. There is therefore likely to be a complex interplay between Prx2 activity, hemoglobin oxidative reactivity and the binding of these two proteins to the erythrocyte membrane. We have drawn attention to the parallels seen in the changes in hemoglobin binding to the erythrocyte during ageing and in extreme exercise. If Prx2 is also shown to behave in extreme exercise as it does in ageing, then extreme exercise may provide an excellent *in vivo* and easily accessible model for the study of erythrocyte ageing.

Oxidative stress, leading to hemichrome formation [14], is known to shorten erythrocyte lifespan [79]. The present study provides the first direct, mechanistic link between oxidative stress, hemoglobin binding to the membrane and the formation of the hemichromes associated with cell senescence. Furthermore we show that hemoglobin binding can be initiated by the oxidative stress induced by the hemoglobin protein alone, even in the absence of any external stressor. We propose that the maximum lifetime of the erythrocyte is therefore set by a timer associated with the redox reactivity of its most abundant protein. This clock likely runs faster in stored erythrocytes, where the antioxidant defenses are compromised and conditions favoring hemoglobin oxidative reactivity, such as a lower pH [80] are prevalent.

## Ethics

This work has been carried out in accordance with the Declaration of Helsinki (2013) of the World Medical Association. It has been approved by the University of Essex Review Process. All blood donors and exercise participants were volunteers and gave informed consent.

## Figures and Tables

**Table 1:** Hb bound to membranes as % of total protein present for different oxidation and ligand binding states of Hb used in the incubation.

Hemoglobin used in incubation	Hb bound as % of total protein present	p compared to oxyHb
(oxidation state of Hb)		
oxyHb (II)	0.07±0.02	–
ferric (III)	0.49±0.07	<0.01
ferryl (IV)	2.1±0.2	<0.01
ferryl with radical (V)	8.8±0.5	<0.01
HbCO (II)	0.08±0.02	0.45
HbCN (III)	0.05±0.02	0.29

The amount of Hb bound to membranes is given as % of the total protein present in the ghosts. Mean±SD; unpaired two-tailed *t*-tests used for comparisons against oxyHb (n=4)

**Table 2:** Proteins detected in membrane protein complex using mass spectrometry following co-IP using anti-Hb antibody.

Protein	Mass spec intensity	Mass spec intensity	p value v. no Hb	Mass spec intensity	p value v. no Hb	Mass spec intensity	p value v. no Hb	Mass spec intensity	Mass spec intensity
	Hb not present in membrane incubation (×10^8^)	Membranes treated with HbCO (×10^8^)		Membranes treated with ferric Hb (×10^8^)		Membranes treated with ferric Hb and H_2_O_2_ (×10^8^)		Membranes treated with ferric Hb (×10^8^)	Membranes treated with ferric Hb and H_2_O_2_ (×10^8^)
								**Normalised to β Hb**	**Normalised to β Hb**
α-spectrin	1.9±0.6	1.6±1.3	0.69	16±2.0	<0.0003	4.7±2.1	0.09	1.2±0.2	0.18±0.1
β-spectrin	0.79±0.35	0.32±0.24	0.13	5.1±1.1	<0.003	1.6±0.8	0.18	0.38±0.1	0.053±0.04
β-hemoglobin	0.0±0.0	7.3±7.0	0.14	11±1.4	<0.002	15±4.2	<0.004	1.0±0.2	1.0±0.3
ankyrin	0.48±0.21	0.17±0.13	0.10	2.7±0.4	<0.0002	0.59±0.30	0.62	0.19±0.05	0.0072±0.007
band 4.2	0.55±0.17	0.50±0.44	0.87	1.8±0.3	<0.002	1.1±0.34	0.06	0.11±0.03	0.037±0.03
α-hemoglobin	0.0±0.0	0.28±0.26	0.19	1.7±1.1	<0.03	1.5±1.4	0.14	0.14±0.09	0.097±0.08

Only proteins that showed significant amounts present following Co-IP in any of the incubations were selected.

Mean intensity±SD (n=3). The p values are shown for unpaired two-tailed *t*-tests relative to incubating membranes in the absence of Hb.
